# Epidemiology and distribution of cruciate ligament injuries in children and adolescents, with an analysis of risk factors for concomitant meniscal tear

**DOI:** 10.3389/fped.2024.1332989

**Published:** 2024-03-08

**Authors:** Xinwang Zhi, Zhicheng Wen, Jiexin Zhang, Dongbo Lai, Huilan Ye, Jianping Wu, Jintao Li, Yan Shao, Federico Canavese, Chun Zeng, Hongwen Xu

**Affiliations:** ^1^ Department of Pediatric Orthopedics, Department of Pediatric Surgery, Guangzhou Women and Children's Medical Center, Guangzhou Medical University, Guangzhou, China; ^2^ Orthopedic Hospital of Guangdong Province, Academy of Orthopedics Guangdong Province, The Third Affiliated Hospital of Southern Medical University, Guangzhou, China; ^3^ Translational Research Centre of Regenerative Medicine and 3D Printing of Guangzhou Medical University, Guangdong Province Engineering Research Center for Biomedical Engineering, State Key Laboratory of Respiratory Disease, The Third Affiliated Hospital of Guangzhou Medical University, Guangzhou, China; ^4^ Department of Pediatric Neurosurgery, Department of Pediatric Surgery, Guangzhou Institute of Pediatrics, Guangzhou Women and Children’s Medical Center, Guangzhou, China; ^5^ School of Pediatrics, Guangzhou Medical University, Guangzhou, China; ^6^ Department of Pediatric Orthopedic Surgery, Lille University Center and Faculty of Medicine, Jeanne de Flandre Hospital, Lille, France

**Keywords:** cruciate ligament, rupture, meniscal tears, children, associated injury

## Abstract

**Introduction:**

To investigate the epidemiological features and prevalence of cruciate ligament injuries (CLI) in children and adolescents, and to examine the potential risk factors associated with concomitant meniscal tear (MT) among this population.

**Methods:**

The demographic data and injury details of children and adolescents with CLI from Southeast China were analyzed to describe their distribution characteristics, alongside an analysis of the prevalence of MTs, the most frequent complication. In addition, binary logistic analysis was employed to ascertain the risk factors linked to MT in individuals suffering from CLI.

**Results:**

A total of 203 patients with CLI (*n* = 206) met the inclusion criteria, with a male-to-female ratio of 2.3:1. Notably, a higher proportion of females were aged ≤16 years old compared to males, who predominated in patients aged >16 years (*P* = 0.001). Among children and adolescents, anterior cruciate ligament (ACL) injuries were the primary type of CLI, accounting for 88.18% (179/203) of all cases. The majority of cases (132/203, 65.02%) were sustained during sports activities, and sprains were the predominant mechanism of injury (176/203, 86.7%). Additionally, the most common associated injury was an MT (157/203, 77.34%). The posterior horn is the most frequently affected site for both medial MT (62.93% out of 73 cases) and lateral MT (70.19% out of 73 cases). Moreover, vertical tears constituted the majority of medial MTs (59.48% out of 116 cases). Furthermore, patients with a higher BMI faced an increased risk of associated MT in comparison to non-overweight patients (88% vs. 73.86%; *P* = 0.038). Each increase in BMI unit was linked with a 14% higher probability of associated MT occurrence in children and adolescents with CLI (OR = 1.140; *P* = 0.036).

**Discussion:**

ACL injuries are a common form of knee ligament injury among children and adolescents, especially those over the age of 16, and are often the result of a sprain. Meniscal posterior horn injury is the most commonly associated injury of youth with CLI. Additionally, overweight or obese people with CLI are at a greater risk of developing MT.

## Introduction

The incidence of cruciate ligament injuries (CLI) has significantly increased in the past two decades, particularly among children and adolescents, due to increased sports participation (1–5). Additionally, there has been a recent sharp rise in anterior cruciate ligament (ACL) reconstruction among patients younger than 15 years old (4, 6). While CLI epidemiological investigation in children and adolescents has been thoroughly researched in Europe and the United States (2, 3, 7, 8), it remains under-researched in Asian countries, particularly China.

The cruciate ligaments are a crucial structure for maintaining proper biomechanics of the knee joint (9). Disruptions of these ligaments can cause immediate and long-term adverse effects. It has been shown that adults who experienced CLI in childhood have a 105 times greater risk of developing osteoarthritis compared to those without such injuries (10). This creates a significant economic burden on society. Furthermore, isolated ACL injuries are infrequent, with other concurrent injuries often present, such as additional ligament injuries, meniscal tears (MT), articular cartilage injuries, and bone injuries (11, 12).

The objective of this study is to investigate the epidemiological characteristics and prevalence of CLI in children and adolescents from Southeast China, as well as to identify the risk factors for concurrent MT in this demographic.

## Methods

This study, conducted at a large tertiary care hospital in Southeast China, aimed to investigate CLI in pediatric patients over a ten-year period from January 2013 to December 2022.

Inclusion criteria consisted of patients diagnosed with traumatic CLI, 18 years of age or younger at the time of injury, and complete medical information (i.e., chart notes, medical history, radiographic records, follow-up notes, etc.).

The study excluded patients with CLI caused by non-traumatic factors, who were older than 18 years at the time of injury, or who had incomplete clinical and radiographic records. To compare the epidemiological characteristics of patients with initial vs. recurrent CLI, we did not exclude patients with a history of previous CLI. However, patients with previous surgery in the surrounding area were excluded.

Demographic information was collected, detailing age, gender, and body mass index (BMI). Additionally, comprehensive data was gathered on cruciate ligament injuries, indicating the affected side, diagnosis (ACL or posterior cruciate ligament), frequency, time of treatment (within or exceeding 8 weeks from injury), mechanism, and causes (sports, traffic accident, and daily life). Additionally, information on associated injuries, such as damage to the meniscus, cartilage, collateral ligaments, and bones, was also obtained.

### Analysis of cruciate ligament injuries

We present a comprehensive profile of CLI distribution using a three-fold approach. This approach allowed for a more objective and precise analysis of CLI distribution among the patient population. In particular: (1) patients' ages were grouped into two categories: those under 16 years old and those over 16 years old; (2) patients were classified as non-overweight (BMI < 24 kg/m^2^) or overweight (BMI ≥ 24 kg/m^2^) according to the Chinese BMI guidelines (13, 14); (3) patients were then divided into two cohorts: acute (patients who received therapy within 8 weeks of their injury) and chronic (patients who received treatment more than 8 weeks after their injury) (15).

### Analysis of concomitant meniscal tears in children and adolescents with CLI

Based on our findings and previous studies (16–18), concurrent MT is the most common complication observed in children and adolescents diagnosed with CLI. This study was designed to investigate in detail the characteristics and distribution of concurrent MT in this population.

**Figure 1:**
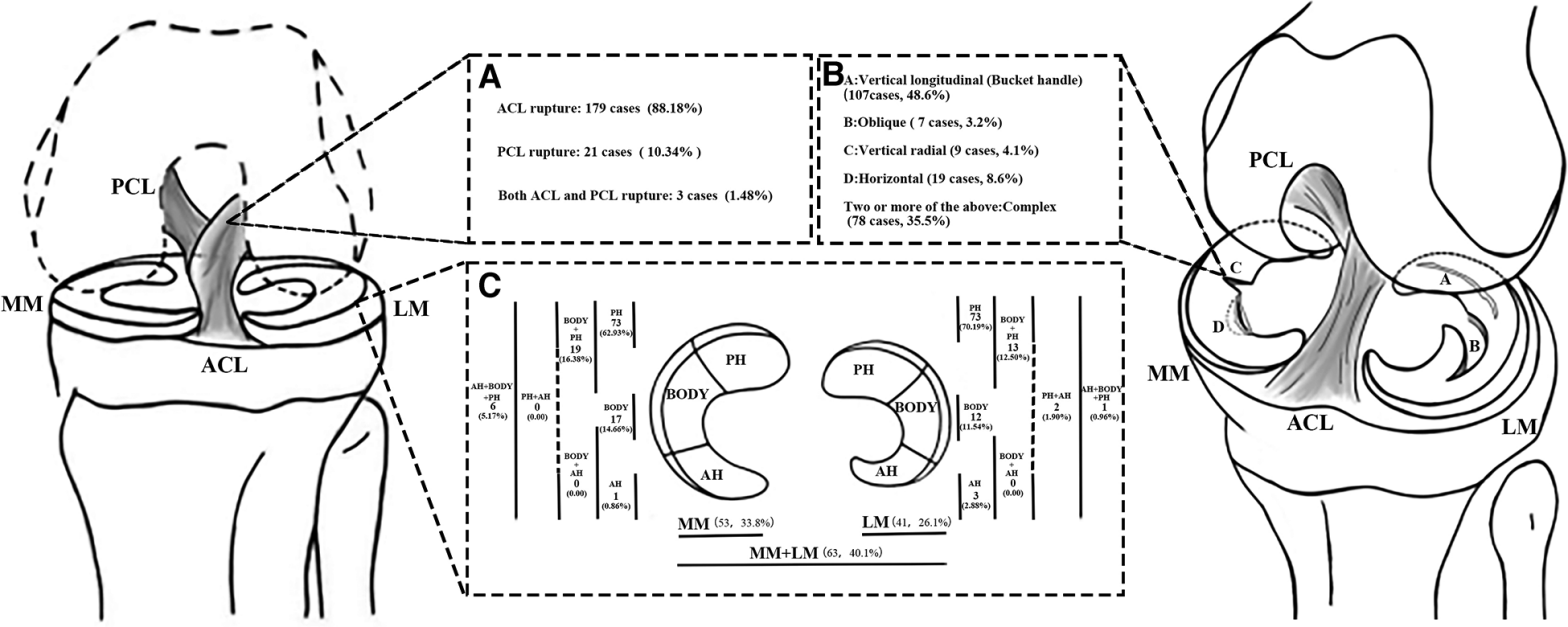
The distribution of cruciate ligament injuries in children and adolescents is presented in (**A**), while (**B**) outlines the classification of meniscal tears according to O’Connor's classification. In (**C**), the meniscus is divided into 1/3 equal parts and the resulting injuries are distributed accordingly. ACL, anterior cruciate ligament; PCL, posterior cruciate ligament; MM, medial meniscus; LM, lateral meniscus; PH, posterior horn; AH, anterior horn.

The investigation focuses on the following aspects: (1) CLI patients with different sex, BMI (non-overweight or overweight), and ages (under or over 16 years old); (2) the different types (ACL or PCL injury), phases (acute or chronic), and frequency (initial or recurrent) of CLI; (3) the medial or lateral MT (M/L MT) location, determined by dividing the meniscus into the anterior horn (AH), posterior horn (PH), and body using the 1/3 equal division method; (4) the types of MT, classified based on tear morphology, include bucket handle (vertical longitudinal), vertical radial, horizontal, oblique, and complex tears (Figure 1).

In addition, this study also examined the risk factors associated with coexisting MTs in children and adolescents diagnosed with CLI from the above aspects.

### Statistical analysis

Statistical analysis was conducted utilizing SPSS 26.0. Descriptive statistics were employed to determine the frequency of various factors investigated. The variables were compared using the Chi-Square Test. Furthermore, binary logistic analysis was utilized to determine risk factors related to MT in patients with CLI. Odds ratios (OR) with 95% confidence intervals were reported for associations. Statistical significance was considered at *P* < 0.05.

## Results

A total of 203 patients with a confirmed diagnosis of CLI (*n* = 206) met the study criteria. Of these, 179 patients (88.2%) had ACL rupture, 21 patients (10.3%) had posterior cruciate ligament (PCL) rupture, and 3 patients (1.5%) had both ACL and PCL rupture. The majority of patients were older than 16 years (*n* = 124, 61.1%), while 79 patients (38.9%) were 16 years or younger. Injury occurred slightly more often in the right knee (*n* = 104, 51.2%) compared to the left knee (*n* = 99, 48.8%) (Table 1).

**Table 1:** Demographic of children and adolescents with cruciate ligament injury.

Socio-demographic characteristics	*N* (%)
Diagnosis	
Anterior cruciate ligament injury	179 (88.18)
Posterior cruciate ligament injury	21 (10.34)
Both	3 (1.48)
Age	
≤16 years	79 (38.92)
>16 years	124 (61.08)
Injury side	
Left	99 (48.77)
Right	104 (51.23)
Gender	
Male	142 (69.95)
Female	61 (30.05)
Body mass index	
Non-overweight	153 (75.37)
Overweight	50 (24.63)
Frequency of injury	
Initial	179 (88.18)
Recurrent	24 (11.82)
Phase of injury	
Acute	110 (54.19)
Chronic	93 (45.81)
Mechanism of injury	
Sprain	166 (81.77)
Fall	20 (9.85)
Crash	7 (3.45)
Others	10 (4.93)
Cause of injury	
Sport	132 (65.02)
Traffic accident	12 (5.91)
Daily life activity	59 (29.06)
Concomitant injury	
Yes	166 (81.77)
No	37 (18.23)

### Gender distribution

**Figure 2:**
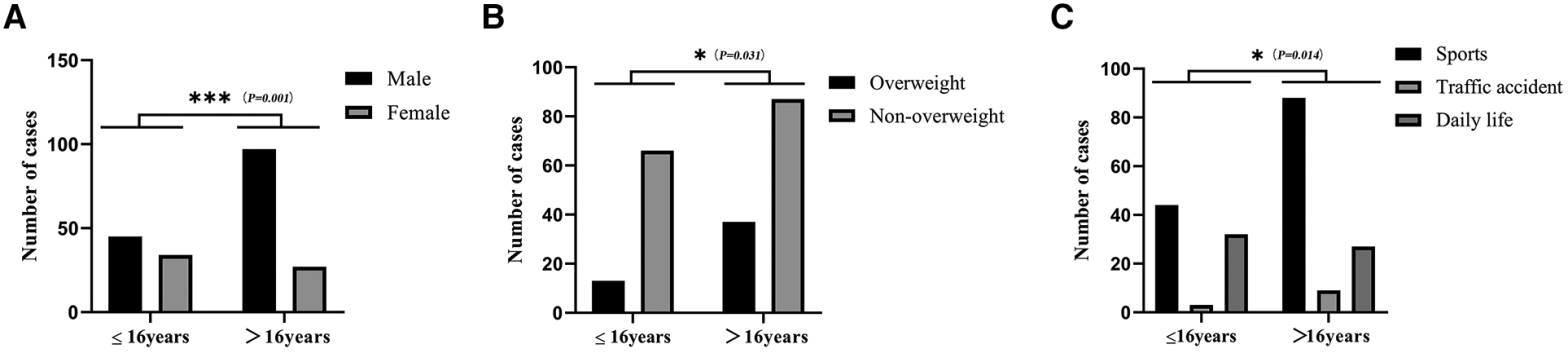
Characterization of cruciate ligament injuries in children and adolescents. Gender distribution of patients with ACL injuries at different ages (**A**), body mass index distribution of ACL injuries at different ages (**B**), and distribution of causes of ACL injuries (**C**) are examined. *P < 0.05; **P < 0.01; ***P < 0.001.

There were more male patients (*n* = 142, 70%) than female patients (*n* = 61, 30%), with an approximate ratio of 2.3:1 (Table 1). In patients younger than 16 years (*n* = 79), 43% were female and 57% were male, whereas in patients 16 years and older (*n* = 124), 21.8% were female and 78.2% were male (*P* = 0.001, Figure 2A).

### Body mass index distribution

According to the BMI criteria for Chinese children and adolescents (13, 14), 75.4% of patients (*n* = 153) were classified as non-overweight and 24.6% (*n* = 50) as overweight (Table 1). Among patients aged 16 years or younger (*n* = 79), 16.5% were overweight and 83.5% were not overweight. However, in patients over 16 years of age (*n* = 124), 29.8% were overweight and 70.2% were normal weight (*P* = 0.031, Figure 2B).

### Mechanism of injury

The majority of cases of CLI were caused by sprains (*n* = 176, 86.7%), followed by falls (*n* = 20, 9.9%), and the least by accidents (*n* = 7, 3.4%). Furthermore, most patients sustained their injuries during sports activities (*n* = 132, 65%), followed by injuries in daily life (*n* = 59, 29.1%) and traffic accidents (*n* = 12, 5.9%) (Table 1). Patients younger than 16 years (*n* = 79) were more likely to be injured during activities of daily living (*n* = 32, 40.5%), whereas patients older than 16 years (*n* = 124) were more likely to be injured during sports activities (*n* = 88, 71%) (*P* = 0.015, Figure 2C).

### Associated injuries

The data show that a minority of patients (*n* = 37, 18.2%) have a single CLI, while the vast majority (*n* = 166, 81.8%) have additional injuries (Table 1). Among those with combined injuries, 157 patients (77.3%) have associated MT, 45 patients (22.2%) have associated cartilage lesions, 12 patients (5.9%) have associated collateral ligament injuries, and 8 patients (3.9%) have associated bone injuries.

### Distribution characteristics of MT in patients with CLI

A total of 157 patients had MT, including 53 patients with MMT, 41 patients with LMT, and 63 patients with bilateral injuries (Figure 1).

The frequency of MT was comparable among CLI patients of different ages and sexes (Table 2).

**Table 2:** Characteristics of associated meniscal tears in children and adolescents with cruciate ligament injury (CLI).

Variable	Meniscus tear (*N*, %)		*χ* ^2^	*P*
Age of CLI patient	YES	NO		
≤16 years	61 (77.22)	18 (22.78)	0.001	0.973
>16 years	96 (77.42)	28 (22.58)		
Gender of CLI patient	YES	NO		
Male	109 (76.76)	33 (23.24)	0.097	0.764
Female	48 (78.69)	13 (21.31)		
BMI of CLI patient	YES	NO		
Overweight	44 (88)	6 (12)	4.302	0.038
Non-overweight	113 (73.86)	40 (26.14)		
Type of CLI	YES	NO		
ACL injury	150 (83.8)	29 (16.2)	38.792	0.000
PCL injury	5 (23.81)	16 (76.19)		
Both	2 (66.67)	1 (33.33)		
Phase of CLI	YES	NO		
Acute	79 (71.82)	31 (28.18)	4.177	0.041
Chronic	78 (83.87)	15 (16.13)		
Frequency of CLI	YES	NO		
Initial	133 (74.3)	46 (25.7)	7.975	0.005
Recurrent	24 (100)	0 (0)		
Side of CLI	YES	NO		
Left	71 (71.72)	28 (28.28)	2.858	0.091
right	85 (81.73)	19 (18.27)		
Type of meniscus tear	Medial meniscus	Lateral meniscus		
Vertical longitudinal tear	69 (59.48)	38 (36.54)	13.424	0.008
Horizontal tear	7 (6.03)	12 (11.54)		
Oblique tear	3 (2.59)	4 (3.85)		
Vertical radial tear	2 (1.72)	7 (6.73)		
Complex tear	35 (30.17)	43 (41.35)		
Site of meniscus tear	Medial meniscus	Lateral meniscus		
Anterior horn	1 (0.86)	3 (2.88)	3.193	0.392
Body	17 (14.66)	12 (11.54)		
Posterior horn	73 (62.93)	73 (70.19)		
Multiple sites	25 (21.55)	16 (15.38)		

**Figure 3:**
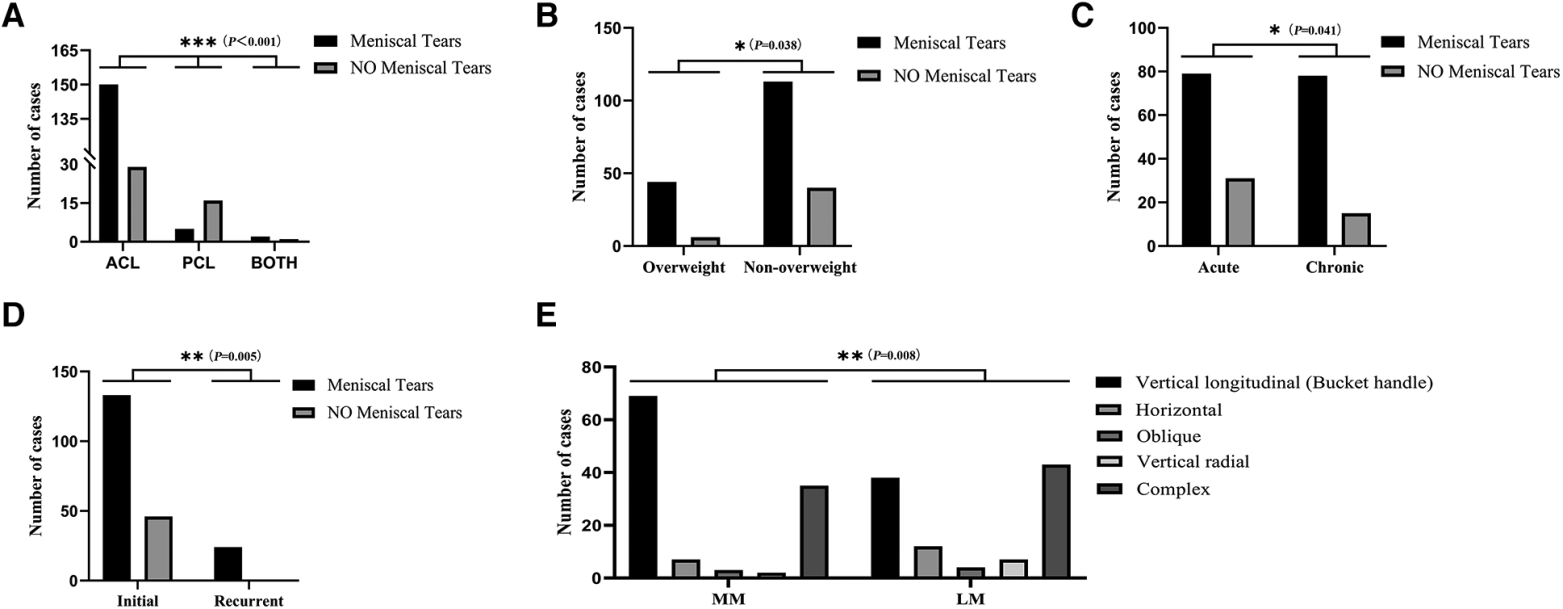
Distribution of concomitant meniscal tears in children and adolescents with cruciate ligament injury. (**A**) Characteristics of meniscal tears in different types of cruciate ligament injury. (**B**) Characteristics of meniscal tears in CLI children and adolescents with different body mass indexes. (**C**) Characteristics of meniscal tears at different stages of cruciate ligament injury. (**D**) Characteristics of meniscal tears in relation to the frequency of cruciate ligament injuries. (**E**) Distribution of different types of meniscal tears. ACL, anterior cruciate ligament; PCL, posterior cruciate ligament; MM, medial meniscus; LM, lateral meniscus. *P < 0.05; **P < 0.01; ***P < 0.001.

ACL injuries were associated with MT in 83.8% of cases, while PCL injuries had an association of 23.81%. Patients with both ACL and PCL ruptures had a 66.7% incidence of MT (*P* < 0.001, Table 2, Figure 3A).

Overweight patients had a higher incidence of MT (88%) compared to their non-overweight counterparts (73.9%) (*P* = 0.038, Table 2, Figure 3B).

In the acute phase, there was a 71.8% association with MT, whereas in the chronic phase, there was an 83.9% association (*P* = 0.041, Table 2, Figure 3C).

Among patients with CLI who experienced a single injury, 74.3% (133 out of 179) had concomitant MT, and 100% (*n* = 24) had concomitant MT among those with a history of re-injury (*P* = 0.005, Table 2, Figure 3D).

The most common location for MMT and LMT in single meniscal injury cases was the PH, with MMT occurring in 73 cases (63%) and LMT in 73 cases (70.2%) (Table 2). Among multiple sites, MT, PH, and body injuries were found to be the most common type. Specifically, the MMT group had 19 cases (16.4%) with injuries at multiple sites, while the LMT group had 13 cases (12.5%).

Of the 116 cases of MMT observed in the distribution of MT types, vertical tear was the most common type, accounting for 59.48% (69/116), while complex tear was the most common type in LMT, accounting for 41.35% (43/104) (*P* = 0.008, Table 2, Figure 3E).

### Risk factors for concomitant MT in children and adolescents with CLI

Binary logistic regression analysis revealed that both ACL injury and BMI were significant risk factors for MT. The ACL injury had an OR value of 16.871 (5.595–50.868, *P* = 0.000), while the OR value of BMI was 1.140 (1.009–1.289, *P* = 0.036), indicating that they are risk factors for the occurrence of concomitant MT in children and adolescents with CLI (Table 3).

**Table 3:** Risk factors for meniscal tear in patients with cruciate ligament injuries.

Risk factors	OR (95% CI)	*P*
Injury time	1.003 (0.998–1.007)	0.243
Body mass index	1.158 (1.108–1.316)	0.025
Type of cruciate ligament injury	15.871 (5.148–48.935)	0.000

## Discussion

This study presents significant findings on the incidence of CLI in pediatric and adolescent populations from Southeast China, coupled with a summary of variables influencing associated MT in these demographics.

Individuals over the age of 16 are more prone to CLI, particularly ACL injuries, reflecting age-related risks, which consistent with prior research (2, 5, 8, 19). The observed phenomenon can be attributed to the anatomical configuration of the ACL in the knee joint. Comparisons demonstrate that PCL is 1.3–2 times thicker and approximately twice as stronger as ACL, thereby providing it with greater resistance against higher forces (20–22). It is possible that changes in stress distribution to the anterior cruciate ligament (ACL) occur after the closure of the epiphyseal growth plate and complete ossification of the tibial epiphysis, which typically happens around the age of 16 (23). Another probable contributor to ACL injuries is the practice of competitive sports like basketball, football, rugby, or similar, which involve sudden stops or turns (19, 24).

Sprains were identified as the leading cause of CLI, accounting for 86.7% of cases. A possible explanation is that young patients have improper movement patterns or inadequate neuromuscular control, leading to hyperextension injuries from valgus or rotational forces (25, 26). In particular, sports activities accounted for 65% of CLI cases. This trend may be related to increased participation in sports, especially among young people (1, 5, 8). Additionally, the increasing prevalence of obesity among adolescents increases the likelihood of injury during sports participation (27, 28). In addition, decreased muscle tone in youth athletes may also play a role in susceptibility to ACL injuries (29).

CLI often results in additional complications, with 81.8% of patients experiencing associated injury in the current study. Furthermore, this risk is particularly notable in males over the age of 16, which is consistent with prior research (4). The increased involvement in high-intensity athletics and greater physical activity, primarily found in teenage males, may be responsible for this occurrence. Furthermore, our research revealed a higher percentage of overweight patients over the age of 16. Modifications to stress dissemination patterns raise stress transference to the cruciate ligament, leading to an increased probability of injuries in those with a high BMI (27). In particular, individuals with higher BMI have a twofold increase in the likelihood of experiencing a combined ACL injury compared to the risk of an isolated ACL injury (16).

The most frequently reported simultaneous injury among children and teenagers with CLI is MT, particularly in the PH of the meniscus, as per our investigations and earlier studies (17, 18, 30). This could be attributed to the higher PH strain as the knee flexion angle increases, even with an intact ACL. When the ACL tears, the restriction of the PH becomes more pronounced, causing damage due to increased stress (31).

In addition, medial meniscus injuries are more common than lateral meniscus injuries, which is consistent with previous research (32, 33). The rotational element common in ACL injuries may significantly contribute to the development of tears in the medial meniscus due to the added strain. Additionally, Allen et al.'s findings indicate that when the knee flexes at a 60° angle, the medial meniscus is subjected to three times the normal stress, increasing the likelihood of MMT occurrence (34).

Vertical tears are the most frequently found type of meniscal tear, particularly in medial meniscus tears, which is consistent with previous studies (19). This is due to longitudinal and radial tears being more common orientations when excessive force is applied to the meniscus, while horizontal tears are more common in degenerative cases (35).

Higher BMI is identified as a risk factor for the development of MT in individuals with CLI. Specifically, overweight patients exhibit a higher susceptibility to MT when compared to non-overweight patients (21). The meniscus serves a critical role in supporting load distribution and shock absorption in the knee joint, while also reducing stress on the articular cartilage and subchondral bone (22, 23). It is believed that the meniscus transmits more than fifty percent of the load in the knee joint, which provides a physiological explanation for the correlation between BMI and MT (24). Consequently, an increased BMI may potentially escalate the strain and pressure in the knee joint during rotation, thereby elevating the risk of developing MT.

The results of our study have significant implications for clinical practice and highlight the need for targeted interventions to reduce the incidence of CLIs and MTs in the youth population. First, the higher prevalence of ACL injuries in youth involved in sports underscores the importance of incorporating injury prevention programs into athletic training programs. In addition, the association between BMI and the likelihood of MT suggests that nutritional counseling and weight management strategies may be beneficial in this population. Clinicians should consider counseling young patients on healthy lifestyle choices as part of their routine care. In addition, our research highlights the need for early and accurate diagnosis of CLI and MT to facilitate timely intervention.

Our study has limitations, as it is retrospective and possibly affected by selection and observational biases. It only comprises patients referred to our institution, and the sample size is small. Therefore, future epidemiologic studies investigating adolescent cruciate ligament injuries with larger samples would be worthwhile. Additionally, this study did not include patients who suffered from cruciate ligament injuries during adolescence but did not receive treatment until adulthood. We also recognize the limitations of BMI as an indicator of body composition, especially in youth athletes who may have increased muscle mass and the need for more nuanced measures in future research. Furthermore, the current study did not explore the treatment of CLI in children and adolescents, which is a future focus of our research.

In conclusion, this study offers significant insight into the typical causes and features of both CLI and injury complications occurring in children and adolescents. ACL injuries are a common form of CLI among children and adolescents, especially those over the age of 16, and are often the result of a sprain. Meniscal posterior horn injury is the most common complication of CLI in youth. Additionally, vertical tears represent the most commonly observed type of MT. Furthermore, individuals with CLI who are overweight or obese are at a higher risk of developing MT. A comprehensive understanding of CLI in this age group could facilitate the creation of successful measures for prevention and targeted interventions, thereby reducing the escalating rates and possible long-term consequences of these injuries.

## Data Availability

The original contributions presented in the study are included in the article/Supplementary Material, further inquiries can be directed to the corresponding authors.
